# Time-Frequency Distribution of Seismocardiographic Signals: A Comparative Study

**DOI:** 10.3390/bioengineering4020032

**Published:** 2017-04-07

**Authors:** Amirtaha Taebi, Hansen A. Mansy

**Affiliations:** 1 Biomedical Acoustics Research Laboratory, University of Central Florida, 4000 Central Florida Blvd, Orlando, FL 32816, USA; hansen.mansy@ucf.edu; 2 Rush University Medical Center, 1653 W Congress Pky, Chicago, IL 60612, USA

**Keywords:** seismocardiography, vibrocardiography, time-frequency analysis, short-time Fourier transform, continuous wavelet transform, chirplet transform, signal processing, signal characteristics

## Abstract

Accurate estimation of seismocardiographic (SCG) signal features can help successful signal characterization and classification in health and disease. This may lead to new methods for diagnosing and monitoring heart function. Time-frequency distributions (TFD) were often used to estimate the spectrotemporal signal features. In this study, the performance of different TFDs (e.g., short-time Fourier transform (STFT), polynomial chirplet transform (PCT), and continuous wavelet transform (CWT) with different mother functions) was assessed using simulated signals, and then utilized to analyze actual SCGs. The instantaneous frequency (IF) was determined from TFD and the error in estimating IF was calculated for simulated signals. Results suggested that the lowest IF error depended on the TFD and the test signal. STFT had lower error than CWT methods for most test signals. For a simulated SCG, Morlet CWT more accurately estimated IF than other CWTs, but Morlet did not provide noticeable advantages over STFT or PCT. PCT had the most consistently accurate IF estimations and appeared more suited for estimating IF of actual SCG signals. PCT analysis showed that actual SCGs from eight healthy subjects had multiple spectral peaks at 9.20 ± 0.48, 25.84 ± 0.77, 50.71 ± 1.83 Hz (mean ± SEM). These may prove useful features for SCG characterization and classification.

## 1. Introduction

Cardiovascular disease is a leading cause of death in the United States, accounting for about 24% of total deaths in 2010 [1]. Improving current diagnostic methods and developing new tools can help decrease associated mortality. Auscultation of heart sounds has been providing useful diagnostic information and is a common test performed during physical examinations. Computer analysis of these sounds can provide additional quantitative diagnostic information that may be helpful for screening patients suspected of heart disease. Seismocardiographic (SCG) signals are the cardiac vibrations measured noninvasively at the chest surface [2,3,4,5,6]. Measurement of cardiac vibrations was performed as early as the start of the 20th century [7]. Many variations within this approach, such as vibrocardiography (VCG), kinetocardiography (KCG), ballistocardiography (BCG), cardiokymography (CKG), and apexcardiography have been described [8,9,10,11,12,13,14,15,16,17,18].

These signals are believed to be caused by the mechanical processes associated with the heart activity (such as cardiac contraction, blood momentum changes, valve closure, etc.). The characteristics of these signals can contain useful information that correlates with cardiovascular pathologies [19]. Such information would also be complementary to other methods that detect the heart’s electrical activity (such as electrocardiography). Early studies [7] suggested that changes in cardiac output may be estimated using these methods. Certain signal patterns were reported in patients with myocardial infarctions [20]. These signals were also found to reflect the strength of myocardial contractions [21,22] and have detectable waveform changes with heart disease resolution [23]. More recent studies suggested utility for monitoring left ventricle function [4,24], and heart and breathing rates [3,9,25,26,27,28]. A case study reported changes in the relative strength of the SCG waves that correspond to valve closure, rapid ventricular filling and ejection that preceded the onset of ischemic symptoms that resolved after therapy [4].

The relation between SCG waves and cardiac activity are not fully understood. However, several studies have investigated that relationship [29,30,31]. For example, SCG was found to contain a low-frequency wave during atrial systole, a high-amplitude wave during ventricular systole, a wave during early ventricular filling, and relatively high-frequency waves at the time of the first and second heart sounds [29]. A three-dimensional model of ventricular contraction indicated that the first SCG peak after electrocardiogram (ECG) R-wave may be related to aortic valve opening [30]. Simultaneous recording of SCG and ECG suggested that the peaks and valleys of the dorso-ventral component of SCG correspond to different physiological events including mitral valve opening and closure, aortic valve opening and closure, isovolumetric contraction, rapid ejection, and rapid filling [31]. Electromechanical systole, pre-ejection period, and left ventricular ejection time were identified using simultaneous recordings of SCG and ECG signals [32]. Multi-channel SCG was used to measure the feature points in a cardiac cycle corresponding to the four valvular auscultation locations. Using this method, six new feature points (including left ventricular lateral wall contraction peak velocity, septal wall contraction peak velocity, trans-aortic valve peak flow, transpulmonary peak flow, trans-mitral ventricular relaxation peak flow, and trans-mitral atrial contraction peak flow) were extracted [33]. However, SCG signals are vulnerable to inter-subject variations such as body mass index, sex, age, and health conditions [3]. In addition, SCG vibrations have relatively low amplitudes that can be easily contaminated by building vibrations, motion artifacts (e.g., patient movements, muscle related disease, etc.) and respiration noise, which can lead to a misinterpretation of SCG signal features [31,34,35,36].

**Figure 1:**
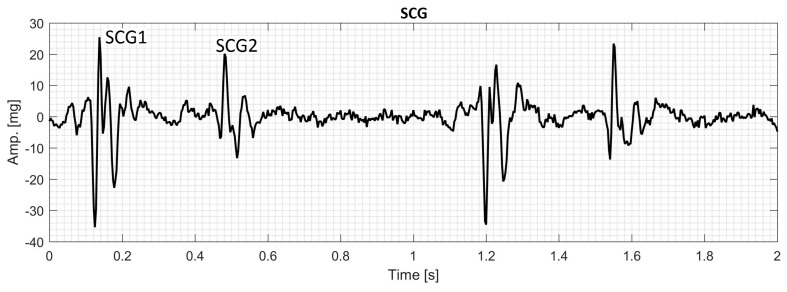
Measured seismocardiographic (SCG) signal showing two cardiac cycles. There are two SCG events (SCG1 and SCG2) for each cycle. These are labeled for the first cycle.

During each cardiac cycle, typical SCG contains two main events that can be called the first SCG (SCG1) and the second SCG (SCG2) as shown in Figure 1. The frequency content of SCG signals is dominated by low frequencies where the human auditory sensitivity is low and may not be sufficient to extract the characteristics of these signals [37,38]. Consequently, examination of SCG signals may not be optimally done by manual auscultation alone and a computer assisted analysis would likely help obtain enhanced qualitative and quantitative description of the signal characteristics. Many methods have been used for SCG analysis [9,10,11,39] including time-frequency analysis [35,40,41]. The current study focusses on extracting spectral features of SCG signals using time-frequency distributions (TFDs).

TFDs have been utilized in the analysis of a wide range of signals including biomedical signals such as electrocardiogram [42,43], electroencephalogram [44,45], phonocardiogram [46,47,48,49], and myoelectric signals [50]. The ability of TFD methods to provide an accurate time-frequency representation depends on the underlying assumptions associated with each method. One common approach for TFD estimation is the short-time Fourier transform (STFT). The STFT is relatively simple, but it may not effectively track steep changes in the time direction [46,51]. In addition, the STFT has static resolution in the time-frequency plane, which can result in resolution limitations. For example, improving the resolution of one of either time or frequency domains worsens the resolution in the other domain. The wavelet transform (WT) was proposed to overcome the resolution issues of the STFT, but the former also has its own resolution limitations [43]. For example, a wavelet shows coarser frequency resolution at higher frequencies and vice versa [52]. This property suggests that WT can be a proper candidate for TFD analysis of signals with discontinuity or steep changes. The wavelet theory can be divided into two important parts; continuous wavelet transform (CWT) and discrete wavelet transform (DWT). While DWT is usually used for signal filtering, denoising and compressing [49,53,54,55,56], CWT is more useful for the signal time-frequency analysis [46,50,57].

The chirplet transform (CT) is another transform that can be viewed as a generalization of both the STFT and WT [58]. It is a transform that involves a function of four parameters: time, frequency, scale and chirp rate; where chirp rate is defined as the instantaneous rate of change in the signal frequency [59]. Since the conventional CT is developed based on the linear chirp kernel, it may provide inaccurate TFD estimations for the signals that have nonlinear instantaneous frequency (IF) trajectory. Therefore, Peng et al. [51] proposed the polynomial chirplet transform (PCT), which is based on a kernel with a polynomial nonlinear IF. This method is more suitable for the signals with IF trajectories that are continuous (either linear or nonlinear) functions of time.

All of the TFDs under discussion are bilinear (or quadratic) representations that combine the time and frequency domain signal information, i.e., they describe the temporal and spectral characteristics of the signal simultaneously. It is worth noting that for a bilinear TFD, the power spectral density (PSD) of a signal that contains two components differs from the summation of the PSD of the components. The cross terms (also called interference terms) in bilinear representations cause artifacts (e.g., extra peaks) in those regions of the time-frequency plane where energy spectral density of the signal components overlap [52].

There are no previous detailed studies that compared the performance of TFD for the analysis of SCG signals. There are, however, studies that addressed similar cardiac signals. For instance, Crowe et al. [43] proposed WT for the analysis of the ECG signals. WT has also been used to analyze ECG signals of patients with some cardiovascular pathology [42]. Obaidat [46] compared the resolution of STFT, WT, and Wigner-Ville distribution for the analysis of phonocardiogram signal. He concluded that WT provided more details of the first and second heart sounds that are acquired by a phonocardiograph. Debbal and Bereksi-Reguig [48] performed a time-frequency analysis of a phonocardiogram signal and compared the performance of STFT, WT and Wigner distribution. Their results suggested that WT revealed the time-frequency characteristics of the signal and was superior to STFT and Wigner distribution in distinguishing between different components of the first and second heart sound. Cherif et al. [49] used wavelet packet transform and discrete wavelet transform with different mother functions to filter murmurs from phonocardiogram signals. They concluded that discrete wavelet transform was more suitable for filtering the murmurs without affecting the heart sound and their components. Also, Ergen et al. [57] investigated the characteristics of the phonocardiogram signals by using wavelets of eight different mother functions and concluded that Morlet was the most appropriate wavelet to extract the features of heart sounds and murmurs.

Understanding different characteristics of SCG, including its TFD, may lead to a more comprehensive description of signals that are related to cardiac activity. Furthermore, successful automatic classification of SCG signals in health and disease can provide possible new methods for diagnosing and monitoring heart function. Time-frequency characteristics are potentially useful features that have been used for automatic classification of similar biomedical signals. However, there is no previous study that focused on determining the most suitable TFD for extracting features of SCG signals.

The objective of the current study is to compare the performances of different TFDs for the analysis of SCG signals. The TFDs that can extract SCG features more accurately will be identified and used to analyze actual SCG signals. A brief description of the theory behind STFT, WT, and PCT is provided in Section 2. Results are presented and discussed in Section 3 followed by conclusions in Section 4.

## 2. Materials and Methods

The TFD of the signals of interest was estimated using six different approaches: short-time Fourier transform, polynomial chirplet transform, and continuous wavelet transform with Daubechies4 (CWT-db4), Coiflet5 (CWT-Coif5), Haar (CWT-Haar), and Morlet (CWT-Morl) as the mother functions. This section provides the definitions and properties of the TFD methods under consideration. Description of the synthetic signals used and the methods of SCG data acquisition is also given.

### 2.1. Time-Frequency Distributions (TFD) Methods

#### 2.1.1. Short-Time Fourier Transform (STFT)

The STFT is obtained by multiplying the signal to be transformed, x(t), by a non-zero window function 𝓌(t). Sliding the window function is then performed to add the time dimension and obtain a time-dependent frequency spectrum. This process can be represented by the equation:

(1) $${\bar{X}}_{STFT}=\int_{-\infty }^{+\infty }x\left(\tau \right)𝓌\left(\tau -t\right)e^{-j\omega \tau }d\tau$$

where 𝓌(t), t, and ω are the window function, time, and frequency, respectively. In this TFD method, the signal x(t) is divided into a number of sub-records that are shorter than x(t). To decrease spectral leakage, the sub-records are multiplied by another window function, and finally, fast Fourier transform is applied to each sub-record. This approach assumes that the signal in each sub-record is stationary. When steep signal non-stationarity is absent in the sub-records, the STFT is expected to provide high-quality estimates of the signal TFD for the whole signal duration. When signal non-stationarity is steeper, the sub-records can be shortened to reduce non-stationarity in individual sub-records and enhance temporal resolution. The shortened time records will, however, worsen the frequency resolution. Hence, refining temporal and spectral resolutions are two competing effects, and a compromise will be needed to reach accurate estimates of TFD.

#### 2.1.2. Continuous Wavelet Transform (CWT)

The CWT of the signal x(t) is defined as follows,

(2) $${\bar{X}}_{WT}=\frac{1}{\sqrt{a}}\overset{+\infty }{\underset{-\infty }{\int }}x\left(\tau \right)\psi^{*}\left(\frac{\tau -t}{a}\right)d\tau =\sqrt{a}\overset{+\infty }{\underset{-\infty }{\int }}X\left(\omega \right)\Psi^{*}\left(a\omega \right)e^{j\omega t}d\omega$$

where *a* is a scale parameter that is inversely related to the frequency. Here, the frequency shifting operation in STFT is replaced by a time-scaling operation in WT. The superscript * denotes the complex conjugate, and ψ(t) is the chosen wavelet mother function. For small and large *a* values, $\psi \left(\frac{\tau -t}{a}\right)$ becomes a contracted and stretched version of the mother function, respectively. Therefore, small and large *a* values may be appropriate for analysis of the high and low frequency components of the signal, respectively. X(ω) and Ψ(ω) are the Fourier transforms of x(t) and ψ(t), respectively. In contrast with STFT that uses the same sliding window at both low and high frequencies, the CWT uses short and long windows at high and low frequencies, respectively. This feature can aid in obtaining better resolution from CWT compared to STFT at low frequencies. Since the scale parameter in the CWT can be considered the inverse of the frequency, the local pseudo-frequency of the CWT may be approximated [60] by:

(3) $$f=\frac{f_{s}f_{c}}{s}$$

where *f* is in hertz, and *f_c_*, *f_s_*, and *s* are the center frequency of the mother wavelet, sampling frequency, and translation parameter (which stands for time), respectively. The center frequencies of the mother wavelets used in the current study are listed in Table 1.

#### 2.1.3. Chirplet Transform (CT) and Polynomial CT (PCT)

In the time-frequency analysis, the chirplet transform of a signal x(τ) can be expressed as [51],

(4) $${\bar{X}}_{CT}\left(t_{0},\omega ,\alpha ;\sigma \right)=\int_{-\infty }^{+\infty }\bar{z}\left(t\right){𝓌}_{\left(\sigma \right)}\left(t-t_{0}\right)e^{-j\omega t}dt$$

where ${𝓌}_{\left(\sigma \right)}$ is a nonnegative, symmetric, and normalized real function. $t_{0}$, ω and α are time, frequency and chirp rate respectively. z(t) is the analytic associate of the signal x(t), and $\bar{z}\left(t\right)$ is defined as,

(5) $$\bar{z}\left(t\right)=z\left(t\right)\Phi_{\alpha }^{R}\left(t\right)\Phi_{\alpha }^{M}\left(t,t_{0}\right)$$

where $\Phi_{\alpha }^{R}\left(t\right)$ and $\Phi_{\alpha }^{M}\left(t,t_{0}\right)$ are the frequency rotating operator and the frequency shift operator respectively and defined as,

(6) $$\Phi_{\alpha }^{R}\left(t\right)=e^{-j\alpha t^{2}/2}$$

(7) $$\Phi_{\alpha }^{M}\left(t,t_{0}\right)=e^{j\alpha t_{0}t}$$

$\Phi_{\alpha }^{R}\left(t\right)$ rotates the analytical associate of the signal by an angle $\theta =tan^{-1}\left(-\alpha \right)$ and $\Phi_{\alpha }^{M}\left(t,t_{0}\right)$ shifts a frequency component from ω to $\omega +\alpha t_{0}$.

For the signals with nonlinear IF trajectory, CT may not accurately track the signal IF [51]. Therefore, the PCT with nonlinear frequency rotating and shift operators and a polynomial kernel is defined to improve the performance of the conventional CT when applied to the signals with nonlinear IF trajectory.

(8) $${\bar{X}}_{PCT}\left(t_{0},\omega ,\alpha_{1},\alpha_{2},\ldots ,\alpha_{n};\sigma \right)=\int_{-\infty }^{+\infty }z\left(t\right)\Phi_{\alpha_{1},\alpha_{2},\ldots ,\alpha_{n}}^{R}\left(t\right)\Phi_{\alpha_{1},\alpha_{2},\ldots ,\alpha_{n}}^{M}\left(t,t_{0}\right){𝓌}_{\left(\sigma \right)}\left(t-t_{0}\right)e^{-j\omega t}dt$$

where $\Phi_{\alpha_{1},\alpha_{2},\ldots ,\alpha_{n}}^{R}\left(t\right)$ and $\Phi_{\alpha_{1},\alpha_{2},\ldots ,\alpha_{n}}^{M}\left(t,t_{0}\right)$ are the nonlinear frequency rotating operator and the frequency shift operator, respectively, and defined as,

(9) $$\Phi_{\alpha_{1},\alpha_{2},\ldots ,\alpha_{n}}^{R}\left(t\right)=e^{-j\sum_{k=2}^{n+1}\;\frac{1}{k}\alpha_{k-1}t^{k}}$$

(10) $$\Phi_{\alpha_{1},\alpha_{2},\ldots ,\alpha_{n}}^{M}\left(t,t_{0}\right)=e^{j\sum_{k=2}^{n+1}\alpha_{k-1}t_{0}^{\left(k-1\right)}t}$$

The PCT can produce a TFD with higher resolution compared to conventional CT for both linear and nonlinear chirp signals [51].

### 2.2. Test Signals

To compare the performance of the different methods in estimating the TFD of SCG, several synthetic signals were generated and analyzed using the above TFD methods. This analysis will provide an estimation of the resolution and accuracy of each method. In this regard, the following synthetic signals were generated. The properties of the generated signals are summarized in Table 2.

#### 2.2.1. Signal with Varying Frequency

The signal consists of one sinusoid (Figure 4a), with an IF law that follows the relation:

(11) $$IF_{1}=-3125\;t^{3}+130.5\;t^{2}+189\;t+18.25\;\left(\mathrm{Hz}\right)$$

where the time vector varies in the range 0≤t≤0.25,

(12) $$x_{1}\left(t\right)=\sin\left(2\pi \left(-781.25\;t^{3}+43.5\;t^{2}+94.5\;t+18.25\right)\left(t+0.1\right)\right)$$

#### 2.2.2. Exponentially Decaying Sinusoid

This signal consists a sinusoid (Figure 5a), with a constant IF of

(13) $$IF_{2}=30\;\left(\mathrm{Hz}\right)$$

(14) $$x_{2}\left(t\right)=1.5e^{-15\left(t-0.1\right)}\sin\left(2\pi \left(30\right)\left(t-0.1\right)\right);\;\mathrm{where}\;0.1\leq t\leq 0.45$$

#### 2.2.3. Decaying Chirp

The signal (Figure 6a) consists of a sinusoid with the IF of

(15) $$IF_{3}=50\left(62.5t^{2}-339.4t+345.5\right)\;\left(\mathrm{Hz}\right)$$

and amplitude of $1.5e^{-15\left(t-0.1\right)}$; where 0.1≤t≤0.45,

(16) $$x_{3}\left(t\right)=1.5e^{-15\left(t-0.1\right)}\sin\left(100\pi \left(\frac{62.5}{3}t^{2}-\frac{339.4}{2}t+345.5\right)\left(t-0.1\right)\right)$$

#### 2.2.4. Double Chirp

The signal (Figure 7a) consists of two chirp components with different amplitudes and IF,

(17) $$IF_{4}=\left(5+\frac{100t}{7}\right)\;\mathrm{and}\;\left(13+\frac{160t}{7}\right)$$

(18) $$x_{4}\left(t\right)=0.7\cos\left(2\pi \left(5+\frac{50t}{7}\right)t\right)+A_{4}\cos\left(2\pi \left(13+\frac{80t}{7}\right)t\right)$$

where 0≤t≤4 and *A*_4_ is defined as follows,

(19) $$A_{4}=0.5+0.1t$$

#### 2.2.5. Growing and Decaying Single Tone with Varying Frequency

The signal (Figure 8a) consists of a quick grow and a slow decay part with the IF of

(20) $$IF_{5}=1.95t^{2}-18t+30.5;\mathrm{where}\;0\;\leq t\leq 4$$

(21) $$x_{5}\left(t\right)=A_{5}\;\sin\left(2\pi \left(0.65t^{2}-9t+30.5\right)t\right)$$

where the signal amplitude is,

(22) $$A_{5}\left(t\right)=\{\begin{array}{ll} \begin{array}{c} -1.82t^{2}+3.55t\;\\ -0.077t^{2}-0.077t+1.58 \end{array} & \begin{array}{c} 0<t\leq 0.65\\ 0.65<t\leq 4 \end{array} \end{array}$$

#### 2.2.6. Synthetic Seismocardiographic (SCG) Signal

In this study, a synthetic SCG signal (Figure 9a) is simulated and described by

(23) $$x_{6}\left(t\right)=\;-A_{6}\sin\left(2\pi \left(20\right)t+94\right)+0.9A_{6}\sin\left(2\pi \left(40\right)t+188\right)$$

It consists of two sinusoids with IF of

(24) $$IF_{6}=20\;and\;40\;\left(\mathrm{Hz}\right),$$

and the signal amplitude varies according to,

(25) $$A_{6}\left(t\right)=\{\begin{array}{c} \begin{array}{cc} 0\; & 0<t\leq 0.25 \end{array}\\ \begin{array}{cc} 0.5-0.5\cos\left(14\pi \left(t-0.75\right)\right)\; & 0.25<t\leq 0.40 \end{array}\\ \begin{array}{cc} 0\; & 0.40<t\leq 0.70\\ 0.45-0.45\cos\left(14\pi \left(t-0.75\right)\right) & 0.70<t\leq 0.83\\ 0\; & 0.83<t\leq 1.00 \end{array} \end{array}$$

### 2.3. Instantaneous Frequency (IF) Error Analysis

The different TFD methods were used to estimate IF of the synthetic signals, which have known IFs. To assess the performance of the different methods, the root mean square error (RMSE) between actual and estimated IF values was calculated as:

(26) $$RMSE=\sqrt{\frac{\sum_{i=1}^{n}{\left(IF_{actual,i}-IF_{estimated,\;i}\right)}^{2}}{n}}$$

where $IF_{actual,i}$ and $IF_{estimated,i}$ are the signal actual and estimated IF at time *i*, respectively. RMSE (root mean square error) values can also be normalized by dividing RMSE by the mean actual instantaneous frequency, ${\bar{IF}}_{actual}$, of each signal such that:

(27) $$NRMSE=\frac{RMSE}{{\bar{IF}}_{actual}}$$

NRMSE (normalized RSME) was used in the current study as a measure of the accuracy of the different TFD techniques in estimating IF, where lower NRMSE values would indicate higher accuracy.

### 2.4. Data Acquisition of Human SCG

**Figure 2:**
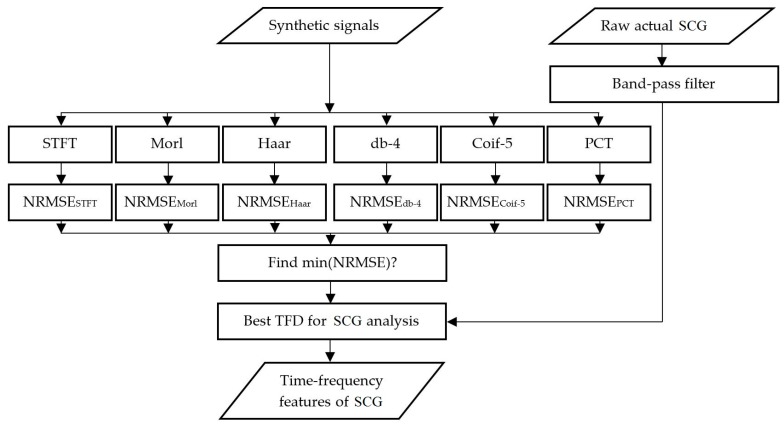
Summary of the signal processing algorithm used in this study.

The above TFD methods were also used to analyze actual SCG signals. The actual SCG signals were measured over the chest of eight healthy volunteers (age: 30 ± 11 years, height: 1.71 ± 0.07 m, weight: 73.66 ± 11.69 kg (mean ± SD)) using a light-weight triaxial accelerometer (X6-2mini, GCDC, Waveland, MS) after IRB approval (at Rush University Medical Center). All participants confirmed that they had no history of cardiovascular disease or disorders. The subjects heart rate was 66.37 ± 2.45 bpm (mean ± SEM) during SCG data acquisition. The sensor was placed at the left sternal border and the fourth intercostal space while subjects were in the supine position. The accelerometer provides a digital signal at a native sampling frequency of 320 Hz. This sampling frequency would be helpful to investigate the higher frequency intra-cardiac events such as heart murmurs and valvular activity as well [61]. The digitized signal was band-pass filtered (0.5–100 Hz) to remove the respiratory noise resulting from breath sounds as well as slow chest wall movement due to breathing [62]. In this study, the dorso-ventral acceleration tended to be stronger than other acceleration components, which agrees with previous studies [31]. Hence, attention in the current study was focused on extracting the TFD of the dorso-ventral acceleration. Matlab (R2015b, The MathWorks, Inc., Natick, MA, USA) was used to process all signals. The overall algorithm is summarized in Figure 2.

## 3. Results and Discussion

**Figure 3:**
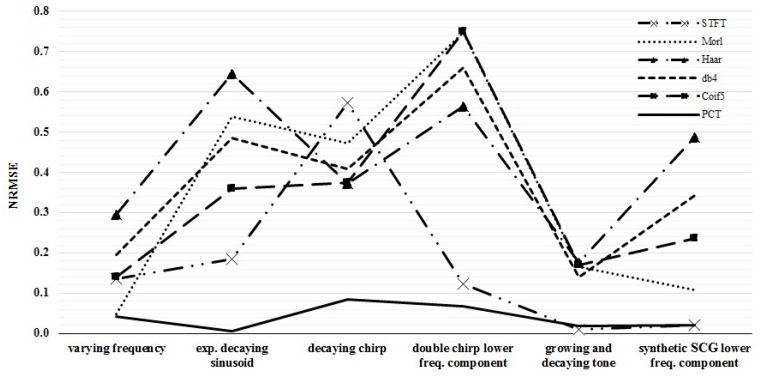
Normalized root-mean-square error (NRMSE) between the actual and calculated IF for different TFD methods. Lower error values would indicate better TFD performance.

Temporal and spectral resolutions of different TFD are shown in Figure 3 and listed in Table 3. The NRMSE between the actual and calculated IF are reported in Table 4 for different TFD. The time and frequency resolutions of each TFD were optimized for each test signal such that the NRMSE was minimized.

As seen in Table 3, STFT had coarser temporal resolution compared to other methods. For example, the temporal resolution was 12.5 ms for STFT and 3.1 ms for all other methods. The frequency resolution for STFT and PCT (at minimum NRMSE) was frequency independent but different for different signals. The resolution mostly ranged from 0.6 to 2.5 Hz and from 0.2 to 0.25 Hz for STFT and PCT, respectively. The spectral resolution (at minimum NRMSE) for CWT-based transforms was frequency dependent (ranging from about 0.4 to 19 Hz for frequencies of 10 to 70 Hz) with finer resolution at lower frequencies. Coarser resolution is undesirable as it may result in higher errors in IF estimation.

**Figure 4:**
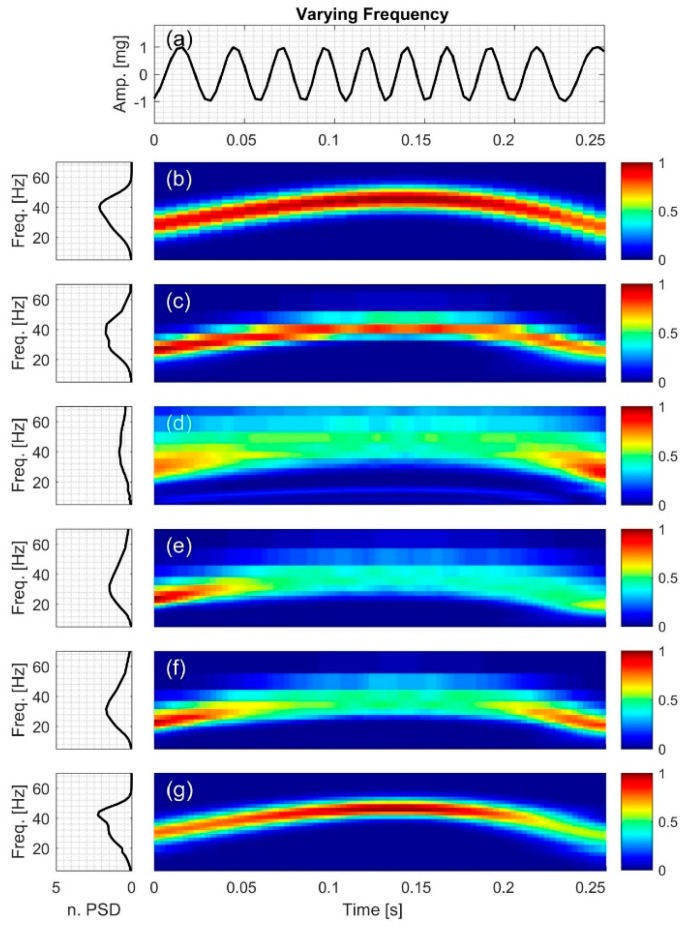
Synthetic test signal with varying frequency: (**a**) Time series. Left and right columns show the power spectral density and time-frequency distribution of the signal using: (**b**) STFT; (**c**) CWT-Morl; (**d**) CWT-Haar; (**e**) CWT-db4; (**f**) CWT-Coif5; and (**g**) PCT, respectively.

**Figure 5:**
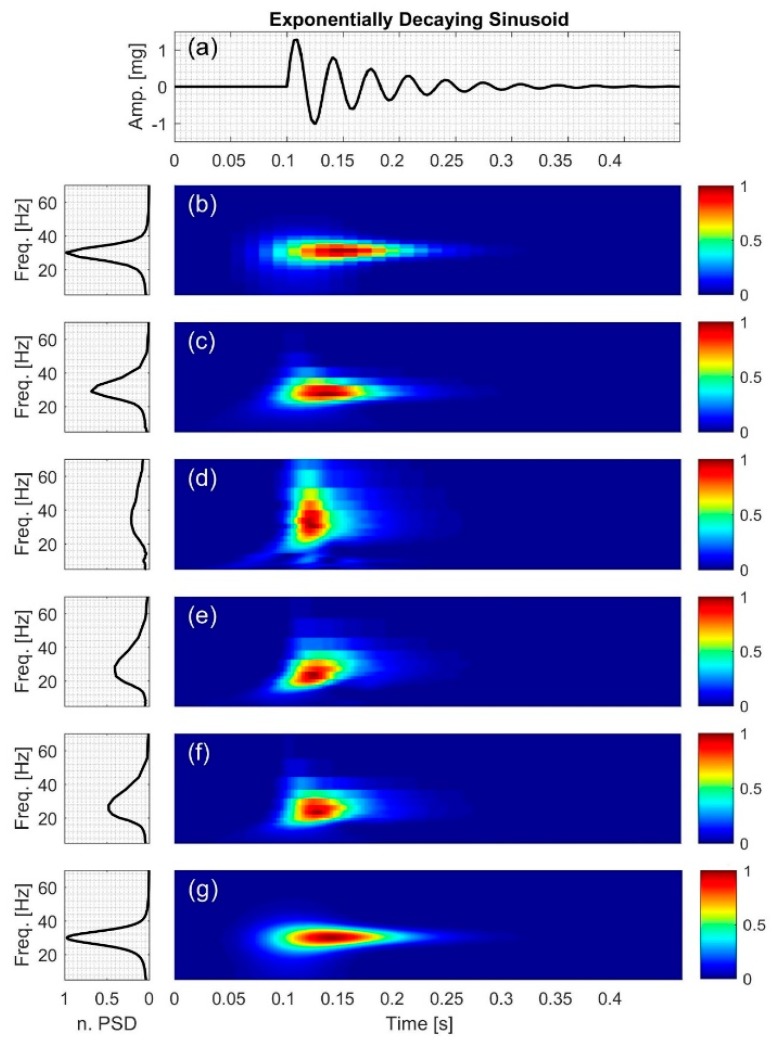
Synthetic test signal with exponentially decaying sinusoid: (**a**) Time series. Left and right columns show the power spectral density and time-frequency distribution of the signal using: (**b**) STFT; (**c**) CWT-Morl; (**d**) CWT-Haar; (**e**) CWT-db4; (**f**) CWT-Coif5; and (**g**) PCT, respectively.

**Figure 6:**
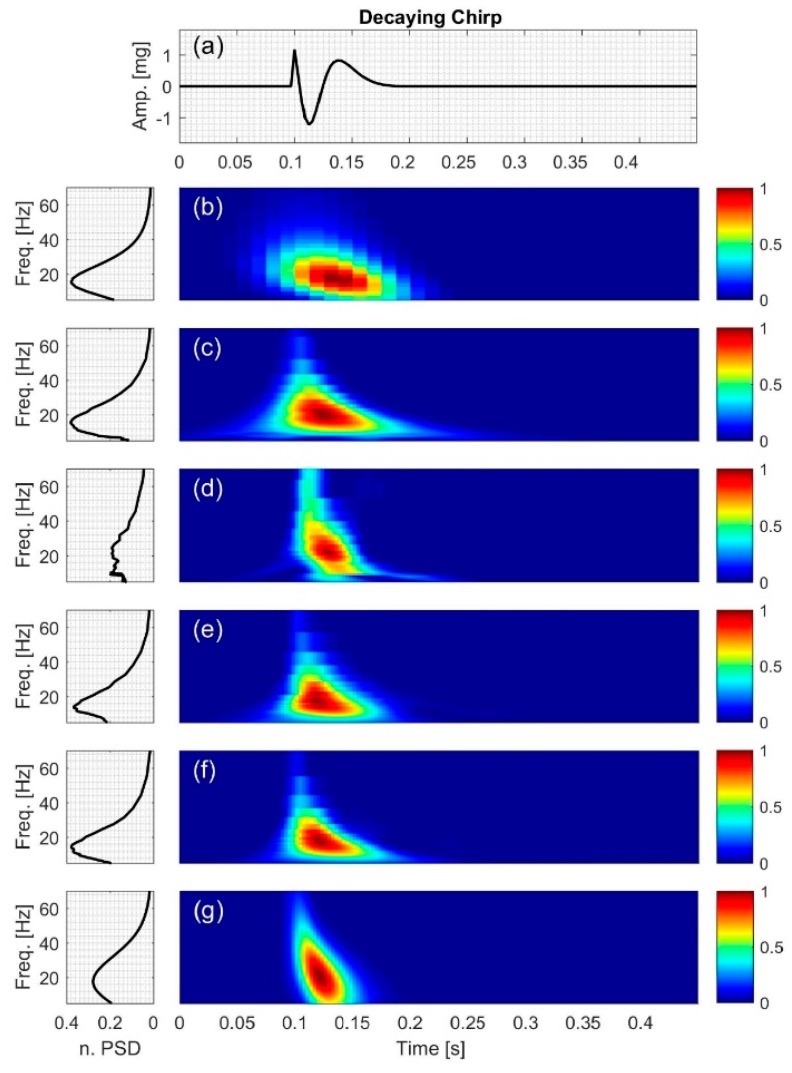
Synthetic test signal with decaying chirp: (**a**) Time series. Left and right columns show the power spectral density and time-frequency distribution of the signal using: (**b**) STFT; (**c**) CWT-Morl; (**d**) CWT-Haar; (**e**) CWT-db4; (**f**) CWT-Coif5; and (**g**) PCT, respectively.

**Figure 7:**
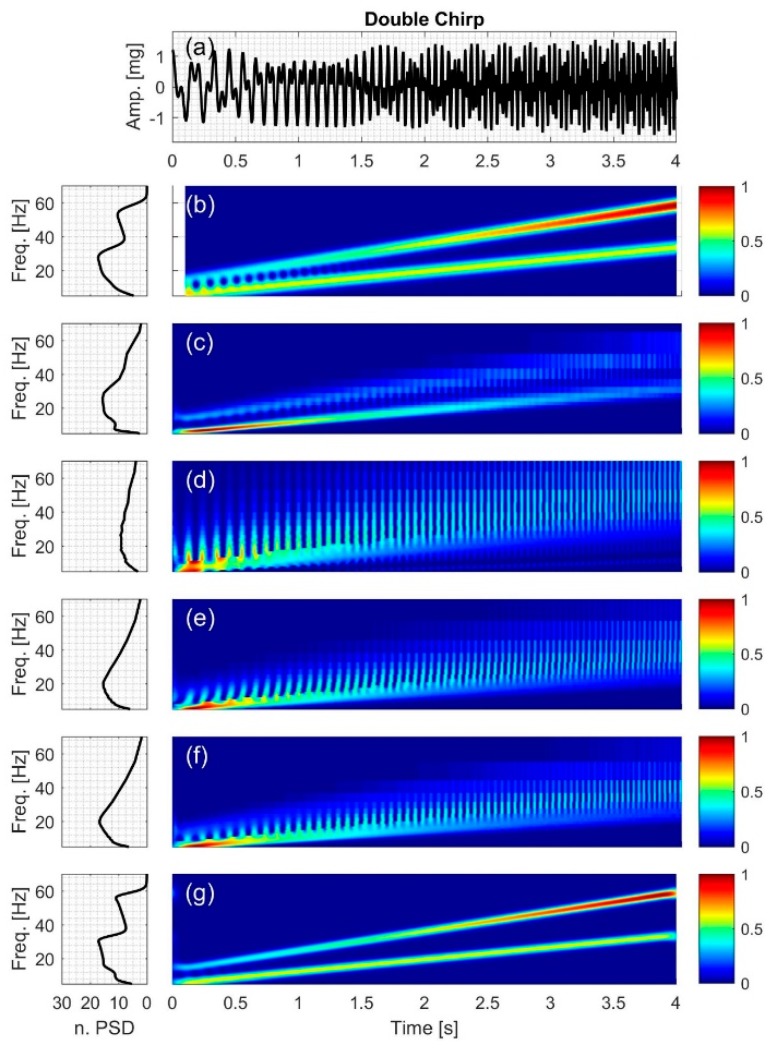
Synthetic test signal with double chirp: (**a**) Time series. Left and right columns show the power spectral density and time-frequency distribution of the signal using: (**b**) STFT; (**c**) CWT-Morl; (**d**) CWT-Haar; (**e**) CWT-db4; (**f**) CWT-Coif5; and (**g**) PCT, respectively.

**Figure 8:**
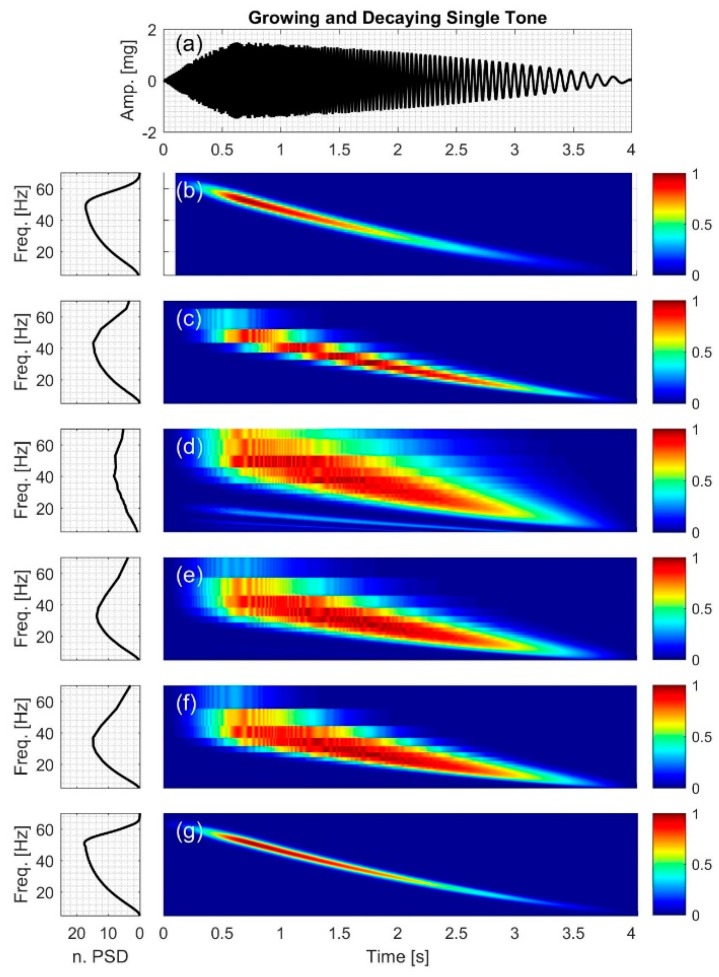
Synthetic test signal with growing and decaying single tone with varying frequency: (**a**) Time series. Left and right columns show the power spectral density and time-frequency distribution of the signal using: (**b**) STFT; (**c**) CWT-Morl; (**d**) CWT-Haar; (**e**) CWT-db4; (**f**) CWT-Coif5, and (**g**) PCT, respectively.

**Figure 9:**
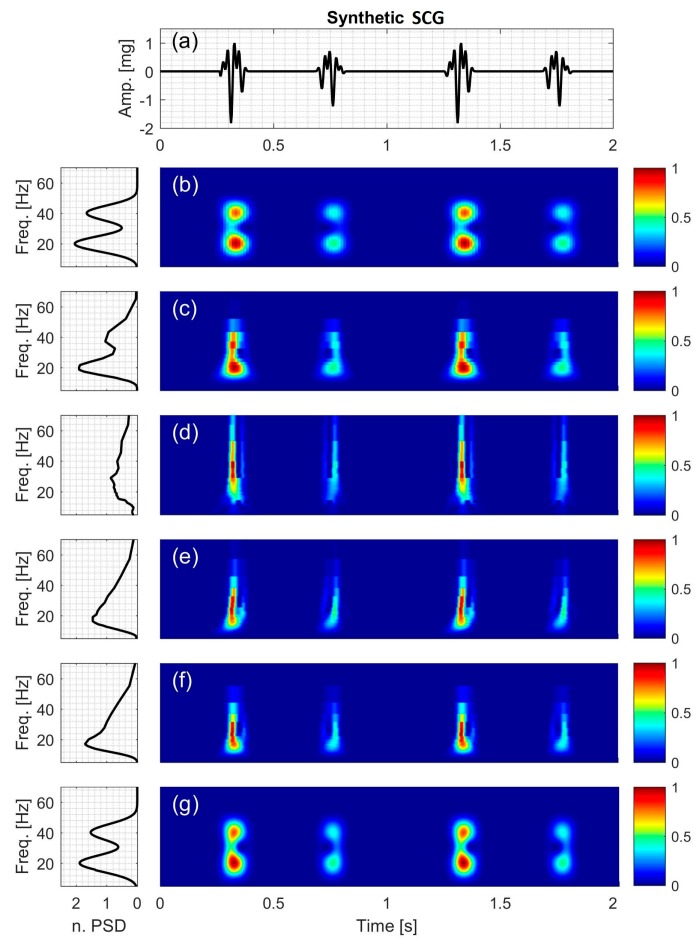
Synthetic SCG signal containing two constant frequency components: (**a**) Time series. Left and right columns show the power spectral density and time-frequency distribution of the signal using: (**b**) STFT; (**c**) CWT-Morl; (**d**) CWT-Haar; (**e**) CWT-db4; (**f**) CWT-Coif5; and (**g**) PCT; respectively.

Figure 4, Figure 5, Figure 6, Figure 7, Figure 8 and Figure 9 show the time series and TFD of the synthetic signals under consideration. The TFD were calculated using STFT (with a Hamming window), CWT-Morl, CWT-Haar, CWT-db4, CWT-Coif5, and PCT in subfigures b, c, d, e, f, and g, respectively. The power spectrum was calculated from the TFDs, normalized with respect to the signal energy, and presented in the left side of Figure 4, Figure 5, Figure 6, Figure 7, Figure 8 and Figure 9. Here, spectral information is shown for frequencies up to 70 Hz as there is no significant energy seen above that frequency.

### 3.1. Signal with Varying Frequency

TFD of the signal with nonlinearly varying frequency, *x*_1_, is shown in Figure 4. Although CWT methods had comparable temporal and spectral resolution, CWT-Morl appeared to have a better TFD estimate than other CWT methods (Figure 4c–f). The different CWT techniques tracked the signal IF with different levels of accuracy. As can be seen in Table 4, the error in estimating IF (among the CWT-based techniques) is lowest and highest for CWT-Morl and CWT-Haar, respectively. CWT-Morl estimated the signal IF more accurately than STFT, although the former had a slightly coarser spectral resolution in the frequency range of this signal. PCT had the lowest error in IF estimation among all methods.

Figure 4 also shows evidence of leakage and edge effects; the CWT-based techniques appear to have more leakage and edge effects compared to STFT and PCT.

### 3.2. Exponentially Decaying Sinusoid

For the exponentially decaying sinusoid, *x*_2_ (Figure 5g), PCT gave the most accurate IF estimation as seen in Table 4. STFT had a lower NRMSE compared to CWT-based methods. CWT-Coif5 estimated the signal IF with less NRMSE compared to other wavelet-based techniques. CWT-Haar appeared to have the highest spectral leakage, which may have affected the power spectral density (PSD) distribution, resulting in a more broad-band PSD than other methods (Figure 5d). On the other hand, PCT and STFT had the lowest leakage as evidenced by the sharper peak in the PSD plots.

### 3.3. Decaying Chirp

For the decaying chirp signal, *x*_3_, PCT estimated the signal IF more accurately than all other methods with an NRMSE value of 0.0850. In addition, CWT-Haar was more accurate than STFT and other CWT-based methods. Figure 6 also indicates that all of the TFD techniques had different levels of leakage as can be seen in their PSD plots. The PSD estimate for CWT-Haar shows more leakage than all methods, although its IF estimate was superior to other CWT-based methods and STFT. The simpler structure of this mother function may have contributed to its increased ability to track time-varying IF (and hence the smaller NRMSE value).

### 3.4. Double Chirp

In double chirp signal (Figure 7), the difference between the two frequency components was chosen to increase with time. All TFD techniques were capable of estimating IF of the lower frequency component but CWT-based methods did not properly capture the higher frequency component, which led to a relatively high NRMSE (0.5651–0.7507) in IF estimation. STFT and PCT had lower error in estimating the signal IF with an NRMSE of 0.1232 and 0.0671, respectively (Table 4).

The ability of the different methods in distinguishing between the two frequency components of the signal varied. For example, by examining Figure 7b, it can be seen that STFT was less successful in distinguishing between the two frequencies’ components at lower frequencies than at higher frequencies.

Interference terms that are common to bilinear methods were also seen. For example, in Figure 7b, the effects of interference terms were clear at lower frequencies where the two spectral components were closer (and consequently have overlapping energies) in the time-frequency plane. At higher frequencies, the two signal components appeared to be sufficiently separated so that cross-term artifacts were not noticeable.

Stronger interference term effects were encountered in CWT methods (Figure 7c–f), which may have hindered, at least in part, their ability to detect the high-frequency component. PCT estimation, on the other hand, resulted in unnoticeable interference term effects at all frequency values, which may have contributed to lowering the NRMSE.

### 3.5. Growing and Decaying Single Tone with Varying Frequency

Figure 8 shows the time-frequency distribution for the growing and decaying single tone with varying frequency, *x*_5_. CWT-based methods had coarser frequency resolution and larger NRMSE compared to PCT and STFT. In addition, CWT TFD appeared to have broader spectral peaks compared to other methods, which is consistent with increased leakage. Figure 8b,g show that PCT had finer resolution in both the time and frequency domain compared to STFT; however, both methods had similar NRMSE (Table 4) and similar PSD.

### 3.6. Synthetic SCG Signal

The synthetic SCG consists of two frequency components at 20 and 40 Hz. Figure 9d–f show that CWT-Haar, CWT-db4 and CWT-Coif5 did not distinguish between the two components with noticeable leakage between them. While CWT-Morl demonstrated less leakage, it showed more leakage compared to STFT and PCT. Consequently, CWT-Morl, STFT and PCT more clearly discriminated between the two signal components compared to the other CWT methods. The PSD graphs of CWT-Haar, CWT-db4 and CWT-Coif5 showed only one peak, while other methods correctly showed two peaks that correspond to the actual signal components.

TFD of the synthetic signals in the current study suggest that PCT consistently had lower NRMSE, less artifacts, and lower leakage. PCT also provided better discrimination between signal frequency components. PCT performance was better than STFT for most synthetic signals, which may be due to the finer resolution of the former. PCT and STFT had more accurate estimations of IF than CWT methods in most cases, especially for signals with multiple frequencies. The performance of CWT methods varied depending on the wavelet mother function and signal under consideration, but did not seem to provide noticeable advantages over STFT or PCT. Among the wavelet mother functions, Morlet had more accurate estimation of synthetic SCG IF, which is consistent with earlier studies of similar signals [57,63]. These trends will be helpful for interpreting the TFD results of actual SCG signals. Since PCT provided more accurate IF values than STFT and CWT, the current study will primarily rely on PCT for estimating the spectral content of actual SCG signals.

### 3.7. Actual SCG Signal

**Figure 10:**
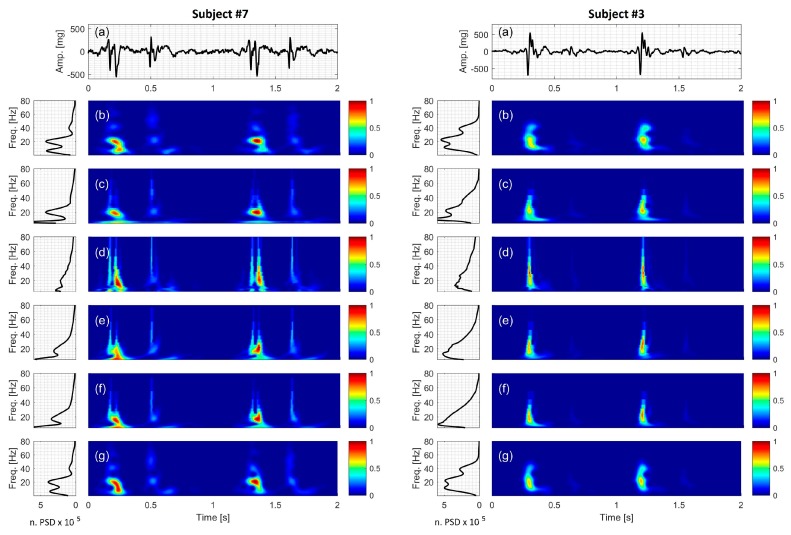
Actual SCG signal of two healthy subjects: (Left two columns) subject #7 (Right two columns) subject #3. For each subject (**a**) Time series. The time-frequency distribution using: (**b**) STFT; (**c**) CWT-Morl; (**d**) CWT-Haar; (**e**) CWT-db4; (**f**) CWT-Coif5; and (**g**) PCT is also shown. The power spectral density for each TFD is shown to the left of the distribution.

TFDs of the dorso-ventral component of actual SCGs are shown in Figure 10 for two cardiac cycles in two different healthy subjects. Data from the rest of the study subjects were similar and are shown in Figure S1 through to Figure S6. In these figures, the spectral information is shown for frequencies up to 80 Hz as there was no significant energy seen above that frequency. Figure 10 suggests that there were two SCG events (which may be called SCG1 and SCG2) for each cardiac cycle. Here, SCG1 and SCG2 appeared to be localized in the time-frequency domain. SCG1 tended to have higher amplitude than SCG2 and different frequency content. While Figure 10 (and Figures S1–S6) showed noticeable inter-subject variability, beat-to-beat variability appeared lower, which is consistent with previous studies [64].

The different TFDs were able to identify SCG spectral peaks with different level of success. For example, PCT and STFT suggested that there were about three dominant frequencies in the actual SCG signal (f_1_, f_2_, f_3_). The peaks related to these frequencies were more clearly seen in the PSD of the STFT and PCT (Figure 10b,g, respectively). However, these peaks were less distinguishable in the spectral estimates of the CWT-Haar, CWT-db4 and CWT-Coif5 (Figure 10d–f), where the PSD showed a broad-band spectrum rather than separate peaks. It is to be noted that although the peak at f_3_ existed in PCT and STFT spectrum for all subjects, it was not always clear in the figures because of its low amplitude. The frequencies at the spectral peaks were calculated using PCT. Calculated frequencies are listed in Table 5 and correspond to f_1_ = 9.20 ± 0.48, f_2_ = 25.84 ± 0.77 and f_3_ = 50.71 ± 1.83 Hz (mean ± SEM). The value of f_3_ suggests that it may be a harmonic of f_2_. However, f_2_ and f_3_ did not appear to be harmonics of f_1_, which suggests that f_2_ and f_3_ may have potentially originated from a source that is different from f_1_. These dominant frequencies take place around the time of strongest SCG wave, which is believed to correspond to ventricular systole [29,31]. Previous studies [65] suggested that the energy associated with frequencies >18 Hz (such as f_2_ and f_3_ in the current study) may be related to valve closure, while lower frequencies (e.g., f_1_ in the current study) are related to heart muscle contraction. Changes in these frequencies may, then, be reflective of ventricular contractility and valve closing. Accurate detection of these changes in the same subject may, therefore, prove useful for monitoring cardiac function.

Comparing Figure 10g with Figure 6g and Figure 9g, one can also conclude that the mid-frequency component, f_2_, of the SCG1 had a slightly decreasing frequency with time (in some subjects). This behavior was not seen for the low and frequency component in most subjects. Figure 10 also shows some SCG beat-to-beat spectral variability that was not clearly demonstrated before. However, further investigation is required to see if these trends are consistent for larger numbers of heartbeats and more diverse subject populations.

**Figure 11:**
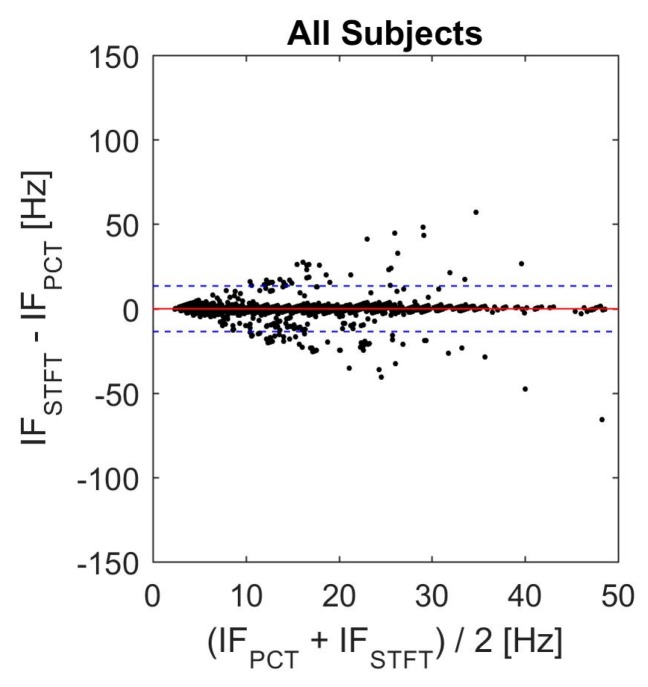
Bland-Altman plot for PCT and STFT IF. The solid line represents the mean (bias) value of differences between instantaneous frequencies. The dashed lines show the 95% confidence interval.

Agreement between the two TFD with the best performance in the current study was assessed using Bland-Altman analysis (Figure 11). In this plot, the solid line represents the mean value of differences between instantaneous frequencies, while the dashed lines show the 95% confidence interval (mean ± 1.96 SD). These values are also listed in Table 6. These results suggest general agreement between PCT and STFT. However, there were some disagreements, which might be due to the inability of STFT to track steep changes in the instantaneous frequency [46,51].

### 3.8. Limitations

The primary limitation of the study is the small number of subjects that participated. Future studies need to be carried out with larger number of subjects from a diverse population covering a wide range characteristics including age, weight, race, and clinical status.

## 4. Conclusions

The objective of this study was to compare the ability of six different approaches in providing accurate time-frequency distribution (TFD) estimates for seismocardiographic (SCG) signals. Methods included short-time Fourier transform (STFT), polynomial chirplet transform (PCT), and continuous wavelet transform (CWT) with four different mother functions. In the current study, the temporal resolution of the STFT was coarser than other methods while the spectral resolution of PCT was the finest among all methods, especially above 10 Hz.

The accuracy of different methods in determining the instantaneous frequency (IF) was tested using synthetic signals with known TFDs, and the estimated IF was compared to actual IF values. CWT performance varied depending on which mother function was used. However, for the simulated SCG signal, the Morlet mother function resulted in more accurate IF values among CWT methods. The errors in estimating IF were lowest for PCT followed by STFT for most test signals. This was particularly true for signals with multiple frequencies including the simulated SCG signal. These results may be attributed to the finer temporal and spectral resolution of PCT and suggest that the method would be a better choice for estimating the TFD characteristics of SCG signals. TFD of actual SCGs was also estimated and PCT results showed that this signal typically had three spectral peaks that tend to be slightly time dependent in some subjects. More studies may be warranted to document the TFD characteristics of SCG signals in health and disease in larger populations.

## Figures and Tables

**Table 1:** Center frequency of the wavelets used in the current study.

Wavelet	Morlet	Haar	Daubechies4	Coiflet5
Center frequency (Hz)	0.8125	0.9961	0.7143	0.6897

**Table 2:** Description of synthetic signals used in the current study.

Signal Description	Frequency Range (Hz)	Peak-to-Peak Amplitude (V)	Signal Length above 5% of Peak to Peak Amplitude (ms)
Varying frequency, $x_{1}$	23 to 45	2.0	250
Exp. decaying sinusoid, $x_{2}$	30	2.3	230
decaying chirp, $x_{3}$	0 to 33	2.4	75
double chirp, $x_{4}^{*}$	7 to 33	3.2	4000
growing and decaying single tone, $x_{5}$	7 to 66	3.0	4000
synthetic SCG, $x_{6}^{*,\#}$	20 and 40	2.8	112

* Signals with more than one dominant frequency component, ^#^ signals with quiet regions.

**Table 3:** Temporal and spectral resolution for different signals and TFD for frequencies between 10 and 70 Hz. The resolution of TFD was optimized to minimize the NRMSE for each synthetic signal. STFT tended to have coarser temporal and spectral resolution compared to PCT. Frequency resolution for CWT-based methods is given as a range with lower values corresponding to lower frequencies.

Resolution	Signal	STFT	Morl	Haar	db4	Coif5	PCT
Temporal resolution (ms)	All signals, $x_{1-6}$	12.5	3.1	3.1	3.1	3.1	3.1
Spectral resolution (Hz)	varying frequency, $x_{1}$	2.5000	0.4000–13.0000	0.3213–15.9377	0.4517–19.0476	0.4361–18.3908	0.2133
	exponentially decaying sinusoid, $x_{2}$	2.5000	0.4000–13.0000	0.3213–15.9377	0.4517–19.0476	0.4361–18.3908	0.2036
	decaying chirp, $x_{3}$	2.5000	0.4000–13.0000	0.3213–15.9377	0.4517–19.0476	0.4361–18.3908	0.2036
	double chirp, $x_{4}$	0.6250	0.4000–13.0000	0.3213–15.9377	0.4517–19.0476	0.4361–18.3908	0.2462
	growing and decaying single tone with varying frequency, $x_{5}$	1.2500	0.4000–13.0000	0.3213–15.9377	0.4517–19.0476	0.4361–18.3908	0.2462
	synthetic SCG, $x_{6}$	0.6250	0.4000–13.0000	0.3213–15.9377	0.4517–19.0476	0.4361–18.3908	0.2462

**Table 4:** Normalized root-mean-square error (NRMSE) between the actual and calculated IF for different TFD methods. Lower error values indicate more appropriate TFD.

Signal	STFT	Morl	Haar	db4	Coif5	PCT
varying frequency, $x_{1}$	0.0248	0.0477	0.2958	0.1954	0.1411	0.0069
exp. decaying sinusoid, $x_{2}$	0.1857	0.5393	0.6463	0.4848	0.3599	0.0056
decaying chirp, $x_{3}$	0.5737	0.4717	0.3733	0.4081	0.3736	0.0850
double chirp, $x_{4}$	0.1232	0.7467	0.5651	0.6612	0.7507	0.0671
growing and decaying single tone, $x_{5}$	0.0109	0.1666	0.1756	0.1393	0.1706	0.0179
synthetic SCG, $x_{6}$	0.0199	0.1084	0.4876	0.3419	0.2370	0.0214

**Table 5:** Dominant frequencies of the actual SCG signals calculated using polynomial chirplet transform. The most dominant frequency for each subject is highlighted in bold.

Subject No.	Heart Rate (bpm)	f_1_ (Hz)	f_2_ (Hz)	f_3_ (Hz)
1	78	**6.15**	22.89	56.12
2	72	11.08	**30.52**	71.38
3	70	11.32	**21.91**	38.65
4	60	8.86	**17.23**	26.09
5	58	5.41	**28.31**	53.17
6	62	**7.63**	32.98	61.05
7	62	6.15	**19.94**	40.12
8	69	16.98	**32.98**	59.08

**Table 6:** Statistical analysis results based on the Bland-Altman method (LOV: level of agreement).

Bias (Hz)	Upper LOV (Hz)	Lower LOV (Hz)
0.7593	17.32	−15.80

## Supplementary Materials

The following are available online at http://www.mdpi.com/2306-5354/4/2/32/s1. Figure S1: Actual SCG signal of subject #1. (a) Time series. The time-frequency distribution using: (b) STFT; (c) CWT-Morl; (d) CWT-Haar; (e) CWT-db4; (f) CWT-Coif5; and (g) PCT. The power spectral density for each TFD is shown to the left of the distribution. Figure S2: Actual SCG signal of subject #2. (a) Time series. The time-frequency distribution using: (b) STFT; (c) CWT-Morl; (d) CWT-Haar; (e) CWT-db4; (f) CWT-Coif5; and (g) PCT. The power spectral density for each TFD is shown to the left of the distribution. Figure S3: Actual SCG signal of subject #4. (a) Time series. The time-frequency distribution using: (b) STFT; (c) CWT-Morl; (d) CWT-Haar; (e) CWT-db4; (f) CWT-Coif5; and (g) PCT. The power spectral density for each TFD is shown to the left of the distribution. Figure S4: Actual SCG signal of subject #5. (a) Time series. The time-frequency distribution using: (b) STFT; (c) CWT-Morl; (d) CWT-Haar; (e) CWT-db4; (f) CWT-Coif5; and (g) PCT. The power spectral density for each TFD is shown to the left of the distribution. Figure S5: Actual SCG signal of subject #6. (a) Time series. The time-frequency distribution using: (b) STFT; (c) CWT-Morl; (d) CWT-Haar; (e) CWT-db4; (f) CWT-Coif5; and (g) PCT. The power spectral density for each TFD is shown to the left of the distribution. Figure S6: Actual SCG signal of subject #8. (a) Time series. The time-frequency distribution using: (b) STFT; (c) CWT-Morl; (d) CWT-Haar; (e) CWT-db4; (f) CWT-Coif5; and (g) PCT. The power spectral density for each TFD is shown to the left of the distribution.
