# Supplementary material for: Time-Frequency Distribution of Seismocardiographic Signals: A Comparative Study
Source: Bioengineering (Basel). 2017 Apr 7;4(2):32. doi: 10.3390/bioengineering4020032 (PMC5590466; doi:10.3390/bioengineering4020032)
Supplement: Supplementary file 1 [file bioengineering-04-00032-s001.pdf]

# Supplementary Materials: Time-Frequency Distribution of Seismocardiographic Signals: A Comparative Study

**Amirtaha Taebi and Hansen A. Mansy**

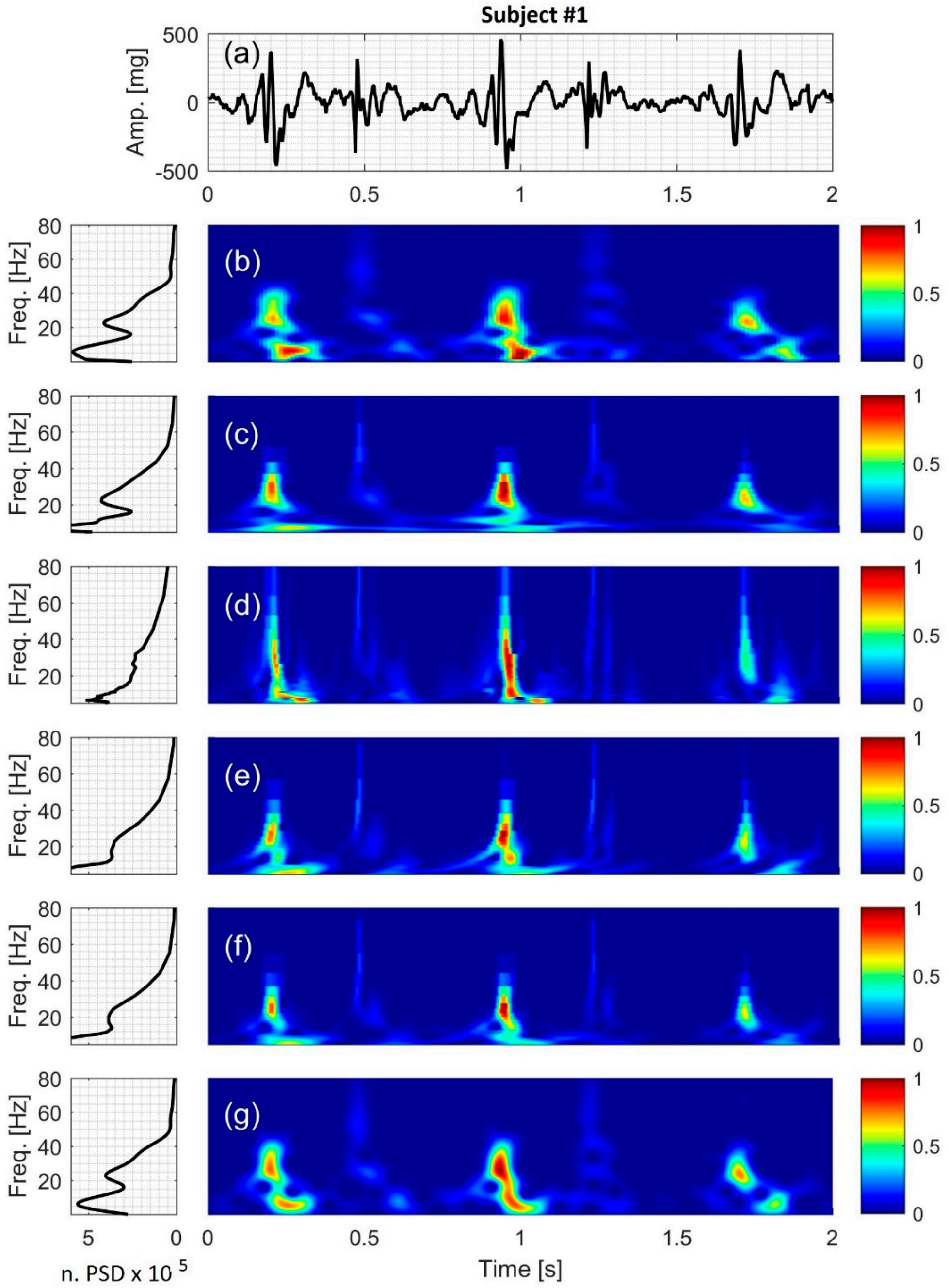


**Figure S1.** Actual SCG signal of subject #1. (**a**) Time series. The time-frequency distribution using: (**b**) STFT; (**c**) CWT-Morl; (**d**) CWT-Haar; (**e**) CWT-db4; (**f**) CWT-Coif5; and (**g**) PCT. The power spectral density for each TFD is shown to the left of the distribution.

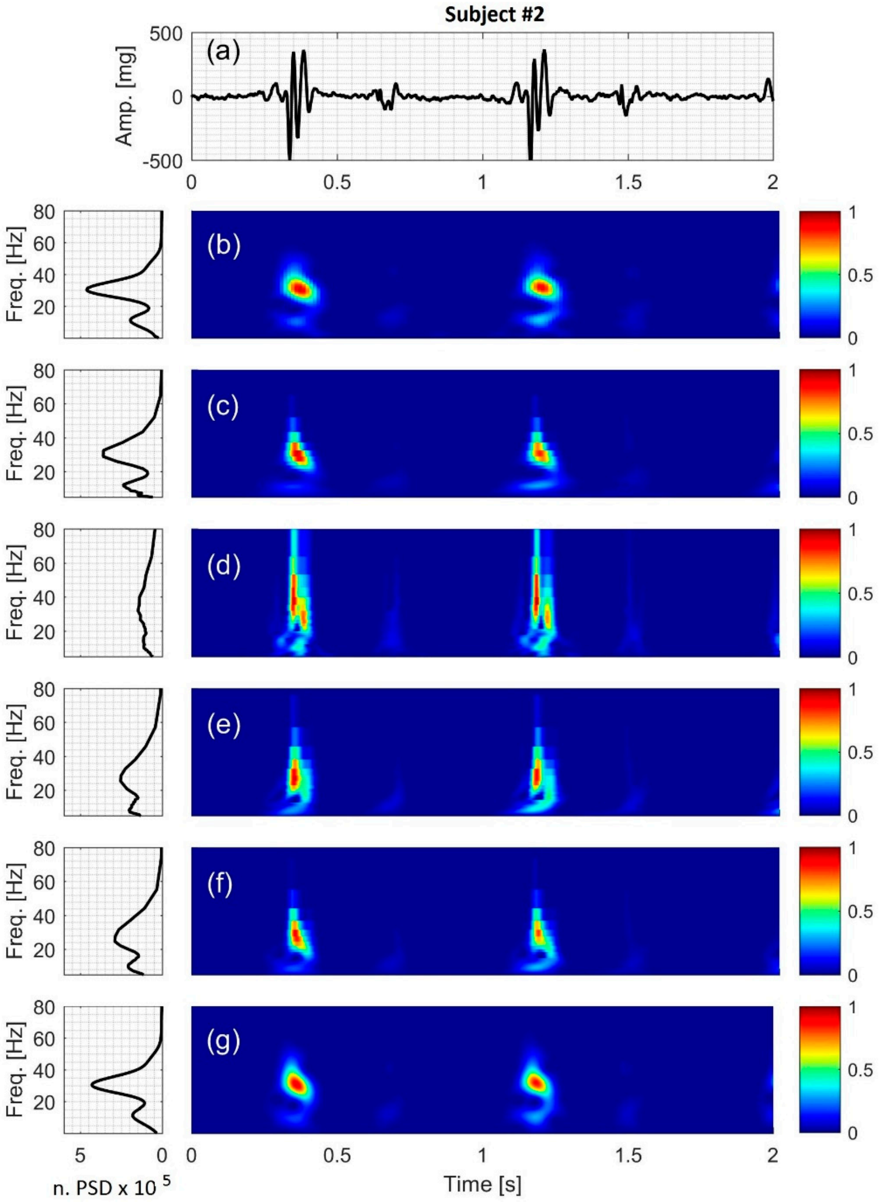


**Figure S2.** Actual SCG signal of subject #2. (**a**) Time series. The time-frequency distribution using: (**b**) STFT; (**c**) CWT-Morl; (**d**) CWT-Haar; (**e**) CWT-db4; (**f**) CWT-Coif5; and (**g**) PCT. The power spectral density for each TFD is shown to the left of the distribution.

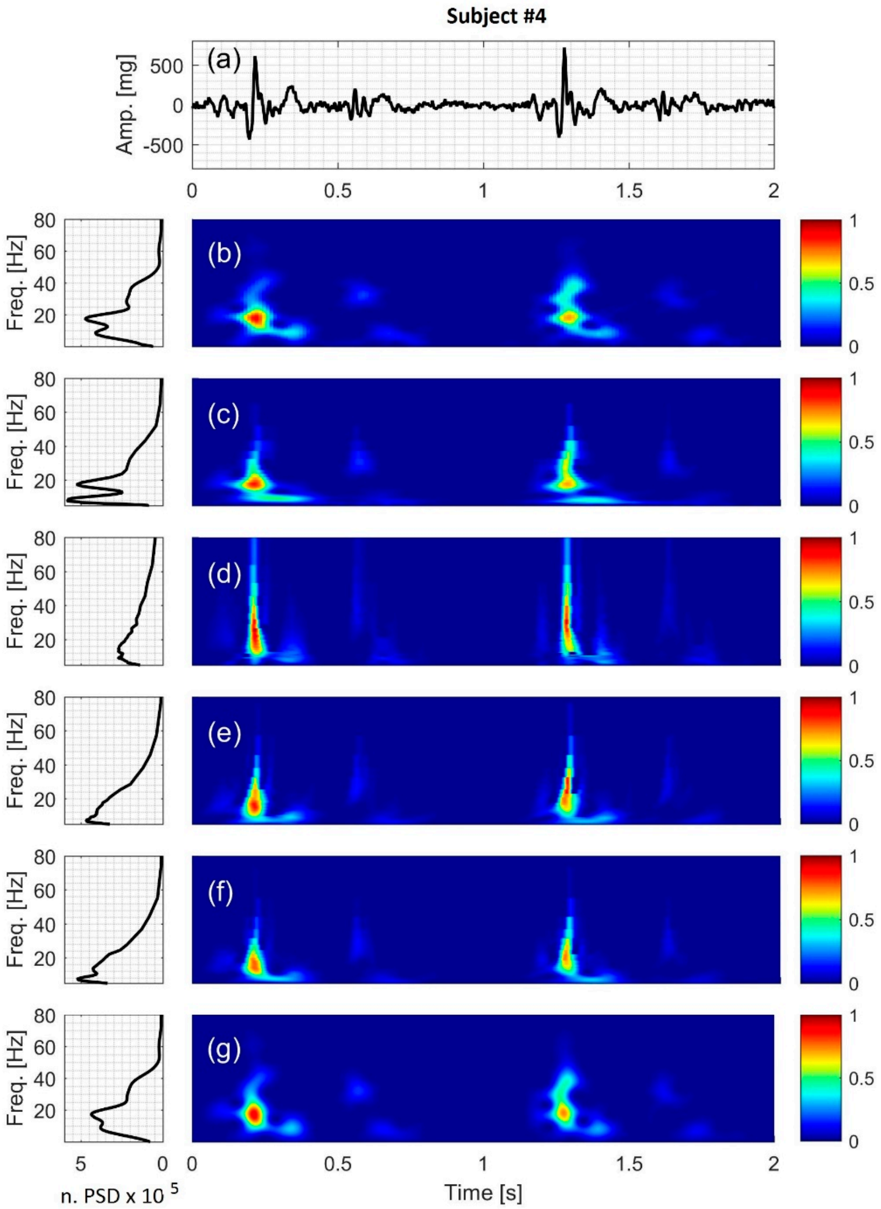


**Figure S3.** Actual SCG signal of subject #4. (**a**) Time series. The time-frequency distribution using: (**b**) STFT; (**c**) CWT-Morl; (**d**) CWT-Haar; (**e**) CWT-db4; (**f**) CWT-Coif5; and (**g**) PCT. The power spectral density for each TFD is shown to the left of the distribution.

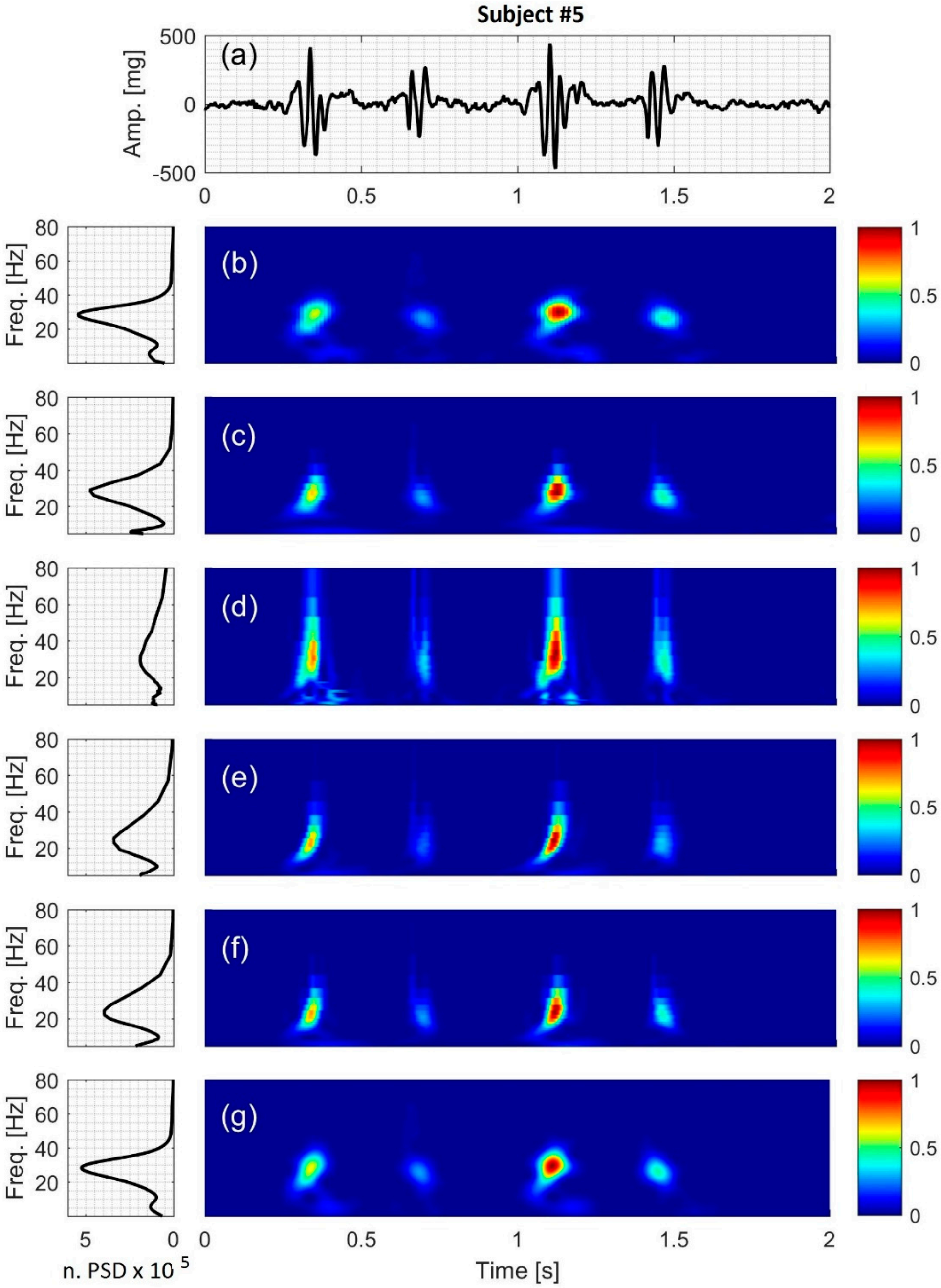


**Figure S4.** Actual SCG signal of subject #5. (**a**) Time series. The time-frequency distribution using: (**b**) STFT; (**c**) CWT-Morl; (**d**) CWT-Haar; (**e**) CWT-db4; (**f**) CWT-Coif5; and (**g**) PCT. The power spectral density for each TFD is shown to the left of the distribution.

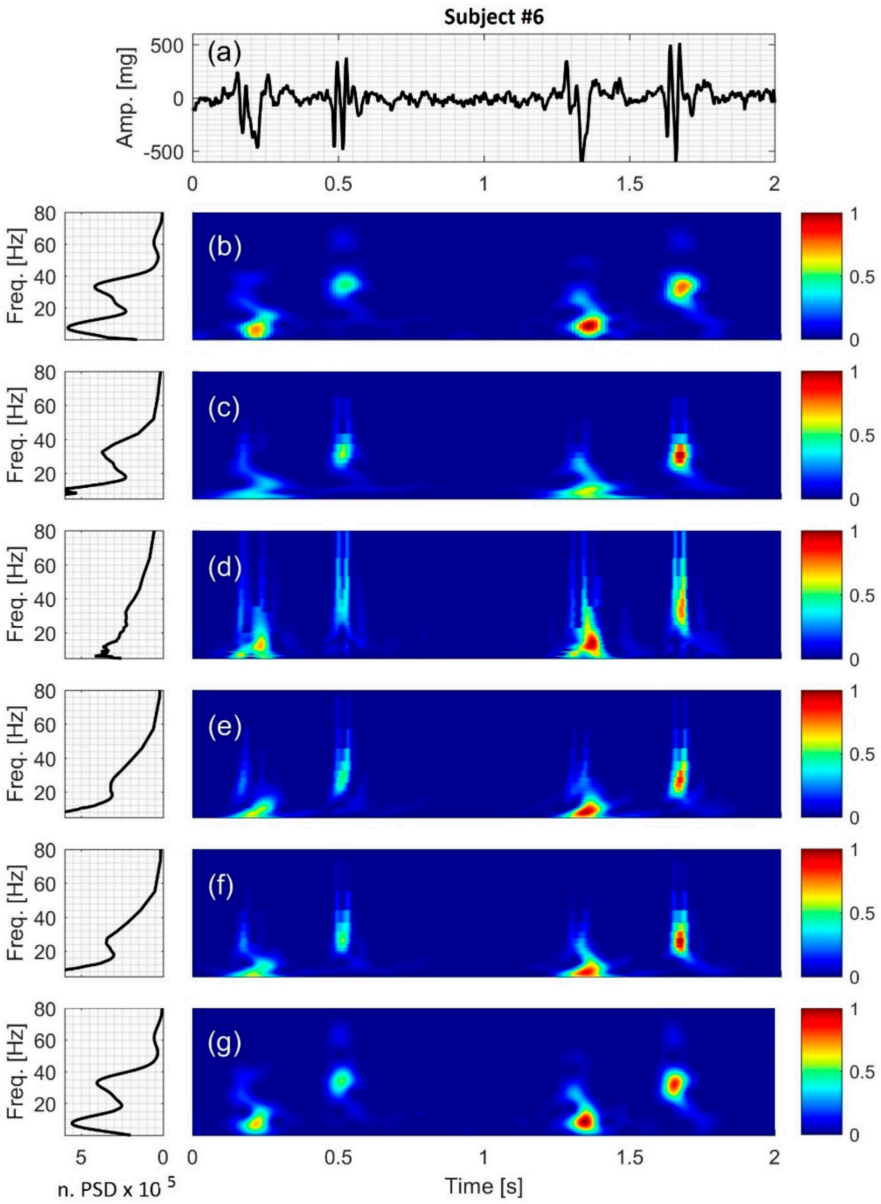


**Figure S5.** Actual SCG signal of subject #6. (**a**) Time series. The time-frequency distribution using: (**b**) STFT; (**c**) CWT-Morl; (**d**) CWT-Haar; (**e**) CWT-db4; (**f**) CWT-Coif5; and (**g**) PCT. The power spectral density for each TFD is shown to the left of the distribution.

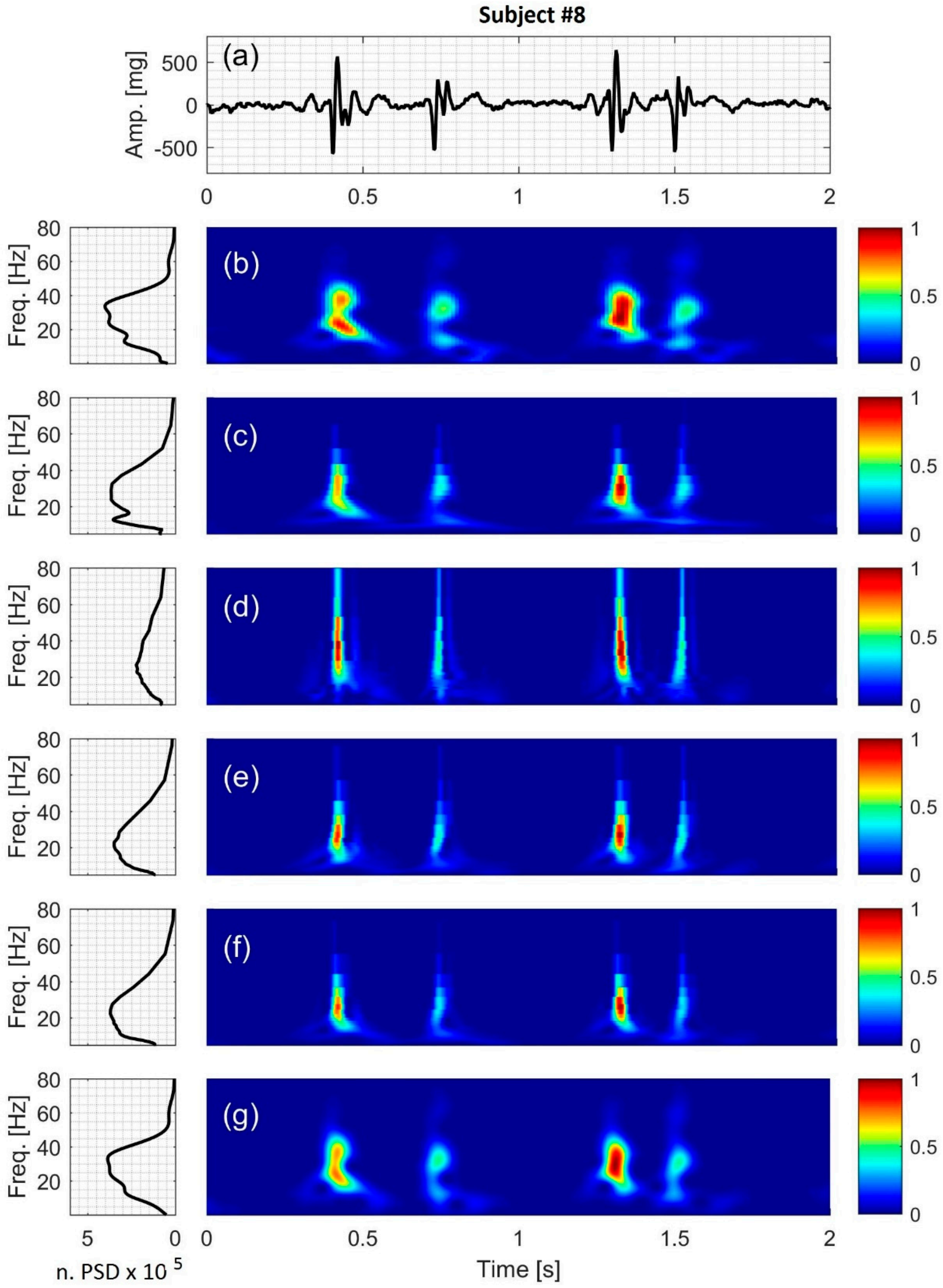


**Figure S6.** Actual SCG signal of subject #8. (**a**) Time series. The time-frequency distribution using: (**b**) STFT; (**c**) CWT-Morl; (**d**) CWT-Haar; (**e**) CWT-db4; (**f**) CWT-Coif5; and (**g**) PCT. The power spectral density for each TFD is shown to the left of the distribution.

© 2017 by the authors. Submitted for possible open access publication under the terms and conditions of the Creative Commons Attribution (CC BY) license (http://creativecommons.org/licenses/by/4.0/).
